# Differential Activation of TRPM8 by the Stereoisomers of Menthol

**DOI:** 10.3389/fphar.2022.898670

**Published:** 2022-06-21

**Authors:** Xiaoying Chen, Lizhen Xu, Heng Zhang, Han Wen, Fan Yang

**Affiliations:** ^1^ Department of Biophysics, Kidney Disease Center of the First Affiliated Hospital, Zhejiang University School of Medicine, Hangzhou, China; ^2^ DP Technology, Beijing, China; ^3^ Alibaba-Zhejiang University Joint Research Center of Future Digital Healthcare, Hangzhou, China

**Keywords:** menthol, stereoisomers, TRPM8, electrophysiology, gating

## Abstract

The stereoisomers of menthol elicit cooling sensation to various levels. Though the high-resolution three-dimensional structures of the menthol receptor, the transient receptor potential melastatin 8 (TRPM8) ion channels, have been revolved in different states, the menthol-bound state structure is not determined and how the stereoisomers of menthol interact with TRPM8 remains largely elusive. Taking advantage of the identical atom composition but distinct spatial orientation of chemical groups in menthol stereoisomers, we performed thermodynamic mutant cycle analysis (TMCA) with patch-clamp recordings to probe the interaction between these ligands and TRPM8. By comparing (−)-menthol with (+)-neoisomenthol or (+)-neomenthol, we observed that the isopropyl or hydroxyl group in menthol interacts with the S4 or S3 helix in TRPM8, respectively. These interactions were also corroborated in our molecular docking of the stereoisomers, though the predicted structural details in the interactions of these ligands with TRPM8 residues are different. Therefore, we suggest similar molecular mechanisms of TRPM8 activation by the stereoisomers of menthol, while the binding configuration of an individual stereoisomer is varied.

## Introduction

Menthol in mint is known to elicit a cool sensation. As a terpenoid alcohol, there are three chiral centers within the menthol molecule, leading to eight possible stereoisomers. Among these stereoisomers, the (−)-menthol, which is the most abundant in nature, also exhibits the lowest cooling thresholds in human taste dilution studies (Hopp, 1993; Barel et al., 2009). Some of the other stereoisomers, such as (+)-neoisomenthol, are less intense in cooling sensation with much higher cooling thresholds. As the stereoisomers of menthol are identical in the number, types, and connectivity of atoms, how they could differentially activate their receptor in humans, the transient receptor potential melastatin 8 (TRPM8) ion channel (McKemy et al., 2002; Peier et al., 2002), to cause differences in cooling sensation remains to be explored.

To investigate the menthol-TRPM8 interactions, functional studies such as thermodynamic mutant cycle analysis (TMCA) with patch-clamp recordings are critical. Though several high-resolution three-dimensional structures of TRPM8 have been resolved by cryo-electron microscopy (cryo-EM) (Yin et al., 2018; Diver et al., 2019; Yin et al., 2019), none of the menthol stereoisomers has been directly observed to be complexed with the channel protein. To probe how menthol binds to TRPM8, by combining molecular docking and TMCA, our previous study showed that (−)-menthol binds to the cytosol-facing cavity formed by the S1–S4 in TRPM8, using its hydroxyl and isopropyl groups as “hand” and “legs,” respectively, to grab and stand on TRPM8 (Xu et al., 2020). TMCA has been successfully applied to reveal the interaction between ion channels and peptide toxins (Ranganathan et al., 1996; Yang et al., 2017) and small molecules (Yang et al., 2015; Xu et al., 2020). A prerequisite for TMCA is that the perturbation introduced to the ligand should not be too large to alter the overall binding configuration of the ligand. Therefore, as the stereoisomers of menthol differ only in the orientation of hydroxyl and/or isopropyl groups, we expect that they are suited for TMCA to probe the ligand-protein interaction in TRPM8.

Moreover, because the TRPM8 channel is a polymodal activated by a plethora of stimuli, ligand–protein interactions are also modulated by these stimuli (Zheng, 2013). For instance, membrane depolarization also directly opens this channel. Previous work has demonstrated that the charged residue R842 on the S4 of TRPM8 contributes to the total gating charge in voltage activation (Voets et al., 2007), so as R842 and other residues of the S1–S4 constitute the (−)-menthol-binding pocket (Xu et al., 2020), and transmembrane voltage is expected to alter menthol-TRPM8 interactions. Therefore, in this study to reveal the mechanisms of TRPM8 activation by the menthol stereoisomers, we systematically investigated their interactions by performing TMCA with patch-clamp recordings at either hyperpolarization or depolarization voltages.

## Materials and Methods

### Molecular Biology and Cell Transfection

Murine TRPM8 was used in this study. Mouse TRPM8 channel was used in this study as mouse and human TRPM8 channels are highly conserved in sequence. Specifically, in the S1 to S4 domains where the menthol stereoisomers are bound, the sequence identity is 96.1% (Supplementary Figure S1A). Point mutations were made by Fast Mutagenesis Kit V2 (SBS Genetech). Primers were used to generate point mutations. All mutations were confirmed by sequencing.

HEK293T cells were cultured in Dulbecco’s modified eagle medium supplemented (DMEM) with 10% fetal bovine serum and 1% penicillin-streptomycin solution at 37°C with 5% CO_2_. When cells grow up to 60%–70%, plasmids were transfected with lipofectamine 2000 following the manufacturer’s protocol. Patch-clamp recordings were performed 18–24 h after transfection.

### Chemicals

(−)-menthol (CAS: 2216-51-5) was purchased from BBI Life Sciences; (+)-menthol (CAS: 15356-60-2) and (+)-neomenthol (CAS: 2216-52-6) were purchased from TCI; (+)-isomenthol (CAS: 23283-97-8) was purchased from Phytolab, and (+)-neoisomenthol (CAS: 20752-34-5) was purchased from Toronto Research Chemicals.

### Electrophysiology

Patch-clamp recordings were performed using a HEKA EPC10 amplifier driven by PatchMaster software (HEKA). Patch pipettes were prepared from borosilicate glass and fire-polished to a resistance of 4–6MΩ. A solution (pH 7.25) containing 130 mM NaCl, 0.2 mM EDTA, and 3 mM HEPES was used in both bath and pipette for recording. For whole-cell recording, cells were detached by trypsin and plated on the microscope cover glass for 30–60 min before the experiment. Transfected cells could be identified by green fluorescence. Cells were clamped at +80 mV and −80 mV for 350 ms, respectively, during recording, and the average current in the last 40 ms was performed. All recordings were performed at room temperature (∼25°C).

To perfuse (−)-menthol and other isomers during the patch-clamp recording, a rapid solution changer with a gravity-driven perfusion system was used (RSC-200, Bio-Logic). Each solution was delivered through a separate tube, so there was no mixing of solutions. The pipette tip was placed right in front of the perfusion outlet during recording to ensure the solution exchange was complete.

### Data Analysis

Data from whole-cell recordings were analyzed in Igor Pro (WaveMatrix). EC_50_ values were derived from fitting a Hill equation to the concentration–response relationship. Changes in EC_50_ by point mutation may be caused by either perturbation of ligand binding or channel gating or both. To distinguish these possibilities, the dissociation constant (*K*_d_) for ligand binding is estimated assuming the following gating scheme:

$$C_{0}\overset{K_{d}}{↔}C_{1}\overset{L}{↔}\mathrm{O,}$$

where *L* is the equilibrium constant for the final closed-to-open transition.

The *P*_o_ of mouse TRPM8 activated by (−)-menthol was measured through single-channel recordings as in our previous study (Xu et al., 2020), and *P*_o_ of TRPM8 mutant channels activated by (−)-menthol was estimated from noise analysis through whole-cell recordings. The mean current amplitude (*I*), the squared deviations in current amplitude from the mean value (*σ* ^2^), and the single-channel current (*i*) were measured experimentally from a membrane patch of ion channels. Then, the number of ion channels clamped into that patch (*N*) is determined as

$$N=\frac{I^{2}}{i\cdot I-\sigma^{2}}.$$

The maximum current when each of the ion channel is at the open state with a *P*_o_ of 1 is equal to *i* × *N*. Then, the open probability was calculated as the ratio between the measured macroscopic current and the maximum current calculated by noise analysis.

For TRPM8 activation by other stereoisomers, *P*_o max_ was measured from the concentration–response curve and *L* was determined from *P*_o max_ by *L* = *P*_o max_/(1 − *P*_o max_). Both *K*_d_ and *L* contribute to the measured apparent affinity by the equation EC_50_ = *K*_d_/(1 + *L*).

To perform thermodynamic cycle analysis, *K*_d_ values of four channel–ligand combinations (WT channel, menthol: *K*_d_ _1; Mutant channel, menthol: *K*_d_ _2; WT channel, menthol analog: *K*_d_ _3; Mutant channel, menthol analog: *K*_d_ _4) were determined separately. The strength of coupling was determined by the coupling energy (kT multiplied by *Ln*Ω, where k is the Boltzmann constant and *T* is the temperature in Kelvin). *Ln*Ω is calculated by the following equation:

$$\text{LnΩ}=\text{Ln}\left(\frac{K_{\text{d}}\_1\cdot K_{\text{d}}\_4}{K_{\text{d}}\_2\cdot K_{\text{d}}\_3}\right).$$

### Molecular Docking

The RosettaLigand application (Meiler and Baker, 2006; Davis and Baker, 2009; Davis et al., 2009) from Rosetta program suite version 2019.12 was used to dock ligand to TRPM8. TRP domain is important for ligand gating in TRPM8 as revealed by a previous study (Xu et al., 2020), so this domain is included in our docking experiments. For docking of stereoisomers, the TRPM8 model (PDB ID: 6BPQ) was first relaxed in the membrane environment using the RosettaMembrane application (Yarov-Yarovoy et al., 2006a; Yarov-Yarovoy et al., 2006b; Yarov-Yarovoy et al., 2012), and the model with lowest energy score was chosen for docking of menthol stereoisomers. Menthol stereoisomer conformers were generated using the FROG2 (Miteva et al., 2010) (http://mobyle.rpbs.univ-paris-diderot.fr/cgi-bin/portal.py#forms::Frog2) server before docking.

As menthol stereoisomers bind to the transmembrane region of TRPM8, the molecular docking approach must consider the energetic effects of the lipid membrane. The membrane environment was set up using the RosettaMembrane energy function (Yarov-Yarovoy et al., 2006a; Yarov-Yarovoy et al., 2006b; Yarov-Yarovoy et al., 2012) in an XML style script in RosettaScripts (Fleishman et al., 2011) (Supplementary Methods). The script also allowed us to control the details of docking. A total of 10,000 models were generated for a docking trial of each ligand. To determine the best docking model, these models were first screened with the total energy score (Rosetta energy term name: *score*). Top 1,000 models with the lowest total energy score were selected. They were further scored with the binding energy between menthol stereoisomers and the channel. Binding energy was calculated as the difference in total energy between the menthol-bounded state and the corresponding apo state models. The top 10 models with the lowest binding energy (*interface_delta_X*) were identified as the candidates. The hydrogen bond between menthol stereoisomers and TRPM8 was determined by UCSF Chimera software. The distance of hydrogen bond was measured between the O1 atom in menthol stereoisomers and the hydrogen atom in the sidechain of R842.

### Molecular Dynamic Simulation

Starting from the transmembrane domain (residue ID 733-977) of the modeled closed-state structure, we used the Membrane Builder function (Jo et al., 2007; Jo et al., 2009; Wu et al., 2014) of the CHARMM-GUI web server (Jo et al., 2008; Lee et al., 2016) to embed the protein in a bilayer of 1-palmitoyl-2-oleoyl phosphatidylcholine (POPC) lipids surrounded by a box of water and ions (with a 15-A° buffer of water/lipids extending from the protein in each direction). The system has a dimension of 110 Å × 110 Å × 85 Å and contains a total of ∼93400 atoms, including 15873 water molecules and 219 POPC molecules. To ensure 0.15 M ionic concentration and zero net charge, 54 K^+^ and 42 Cl^−^ ions were added. A menthol-bound system was built following the same settings except one menthol molecule was docked to each subunit as previously described. After energy minimization, six steps of equilibration were performed (with gradually reduced harmonic restraints applied to protein, lipids, water, and ions). Finally, we conducted production MD runs in the NPT ensemble. The Nosé–Hoover method (Nosé, 1984; Hoover, 1985) was used with a temperature of T = 30°C. The Parrinello–Rahman method (Parrinello and Rahman, 1981) was used for pressure coupling. For nonbonded interactions, a 10- A° switching distance and a 12-A° cutoff distance were used. The particle mesh Ewald method (Darden et al., 1993) was used for electrostatics calculations. The LINCS algorithm (Hess et al., 1997) was used to constrain the hydrogen-containing bond lengths, which allowed a 2-fs time step for MD simulation. The energy minimization and MD simulation were performed with the GROMACS program (Pronk et al., 2013) version 5.1.1-gpu using the CHARMM36 force field (Klauda et al., 2010; Huang and MacKerell, 2013) and the TIP3P water model (Jorgensen et al., 1983). The parameters for the menthol molecules were generated with the CHARMM General Force Field (Vanommeslaeghe et al., 2010).

## Results

### Differential Activation of TRPM8 by the Stereoisomers

**FIGURE 1:**
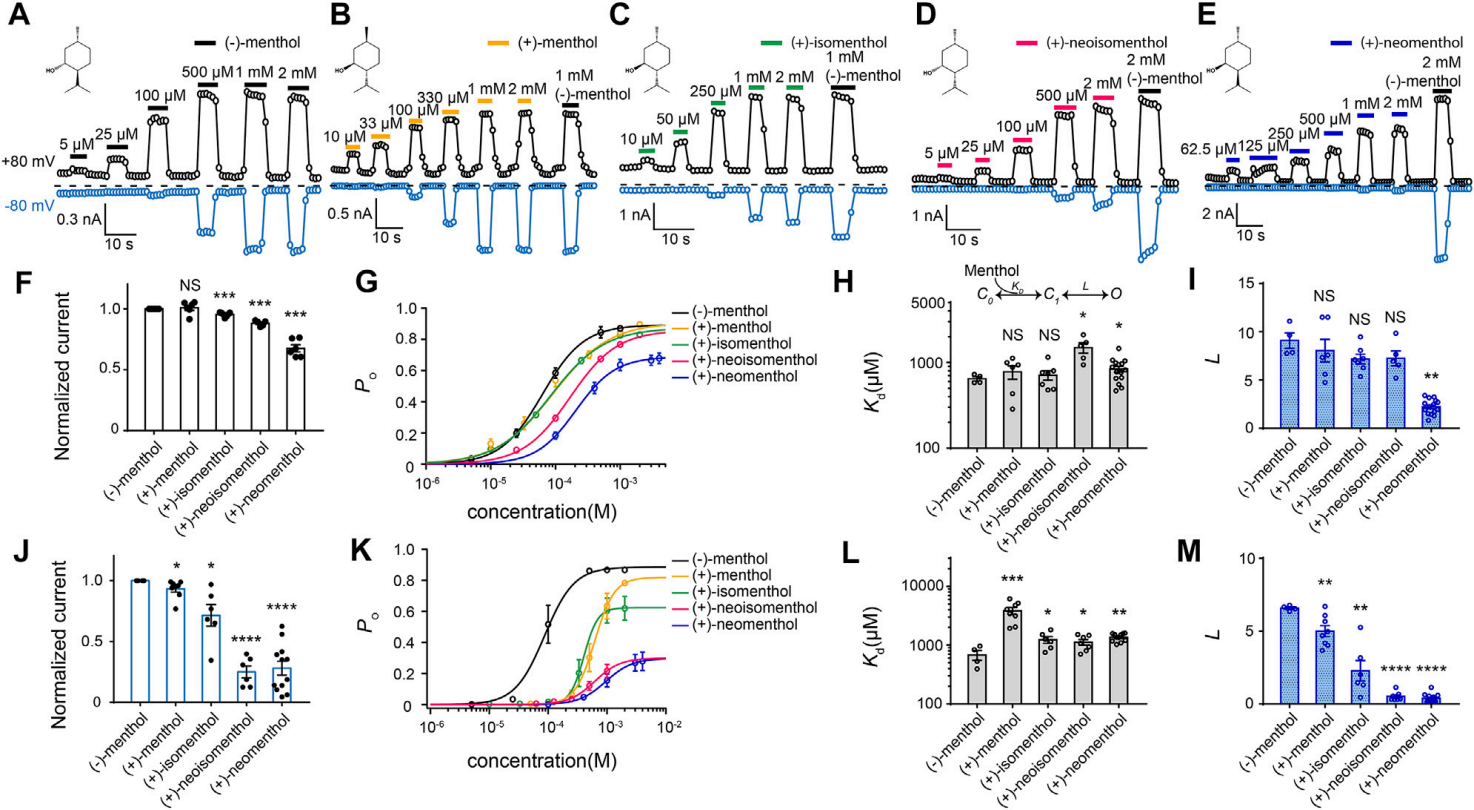
Differential activation of mouse TRPM8 by menthol stereoisomers. **(A–E)** Representative whole-cell recordings of mouse TRPM8 activated by different menthol stereoisomers in a concentration-dependent manner. The chemical structures of menthol stereoisomers are shown on upper left. **(F)** Bar graph of normalized currents induced by menthol stereoisomers at +80 mV from HEK293T cells transfected with mouse TRPM8 recorded in whole-cell configuration (*n* = 4–7 for each isomer, two-sided *t*-test, *p < 0.05; **p < 0.01; ***p < 0.001; ****p < 0.0001; NS, not statistically significant). Stereoisomers invoked max current amplitudes that were normalized to the response to 2 mM (−)-menthol. **(G)** Concentration–response curve of menthol stereoisomers activation measured from whole-cell patch-clamp recordings at +80 mV (*n* = 4–7). **(H,I)** General gating scheme where the ligand binding is represented by *K*_d_, and the equilibrium constant between the closed and open states upon ligand binding is represented by *L*. For different stereoisomers, *K*_d_ and *L* values were calculated from concentration–response curves in **(G)** (*n* = 4–7, two-sided *t*-test, *p < 0.05; **p < 0.01; ***p < 0.001; ****p < 0.0001; NS, not statistically significant). All statistical data are given as mean ± s.e.m. **(J)** Bar graph of normalized currents induced by menthol stereoisomers from HEK293T cells transfected with mouse TRPM8 recorded in whole-cell configuration (*n* = 4–7 for each isomer, two-sided *t*-test, *p < 0.05; **p < 0.01; ***p < 0.001; ****p < 0.0001; NS, not statistically significant). Stereoisomers invoked max current amplitudes that were normalized to the response to 2 mM (−)-menthol. **(K)** Concentration–response curve of menthol stereoisomers activation measured from whole-cell patch-clamp recordings at −80 mV (*n* = 4–7, two-sided *t*-test, *p < 0.05; **p < 0.01; ***p < 0.001; ****p < 0.0001; NS, not statistically significant). **(L,M)** At −80 mV, *K*_d_ and *L* values of different stereoisomers were calculated from concentration–response curves in **(K)**. All statistical data are given as mean ± s.e.m.

We first measured TRPM8 activation by the five commercially available menthol stereoisomers with whole-cell patch-clamp recordings (Figures 1A–E). TRP channels, including TRPM8, are polymodal receptors activated by ligands, depolarization, or temperature. Therefore, when we performed patch-clamp recordings, we kept the recording temperature (∼25°C) and clamping voltage (±80 mV) constant, so that only the concentration of ligand (menthol stereoisomers) was changed. Moreover, at 25°C and −80 mV, the TRPM8 channel was barely activated if no menthol was perfused (Supplementary Figure S1B). Though at 25°C and +80 mV, we observed a small current in the absence of menthol, perfusion of menthol elicited a much larger current. Therefore, the changes in current we measured from patch-clamp recordings were indeed caused by channel activation by menthol stereoisomers but not by other stimuli.

We observed that while all these stereoisomers activated TRPM8 current in a concentration–dependent manner, current activation was differentially modulated by transmembrane voltage. At +80 mV, the maximum current amplitudes induced by the stereoisomers normalized to that of (−)-menthol were similar except for (+)-neomenthol (Figure 1F). Their concentration-dependent curves were also shifted to higher concentrations as compared to that of (−)-menthol, but the changes in EC_50_ values were less than ten-fold (Figure 1G). EC_50_ of (−)-menthol and (+)-neomenthol was 62.64 ± 1.2 µM and 206.22 ± 11.4 µM, respectively) (Figure 1G).

To quantify the ligand–protein interactions, we employed the simple gating scheme (Figure 1H) that successfully described the (−)-menthol and capsaicin binding and activation of TRPM8 (Xu et al., 2020) and TRPV1 (Yang et al., 2015), respectively. In this scheme, *K*_d_ and *L* reflect the binding affinity and gating capability of the ligand, respectively. These parameters were determined from EC50 values and the maximum open probability (*P*_o__max) (see Methods for details). As we have measured the *P*_o__max of wild-type TRPM8 activated by (−)-menthol with single-channel recordings (Xu et al., 2020), we normalized the current amplitude induced by a stereoisomer to that of (−)-menthol to calculate the *P*_o__max of this ligand. In this way, we calculated the *K*_d_ and *L* values of the stereoisomers based on their concentration-dependent curves (Figures 1H,I). Surprisingly, the *K*_d_ values of all stereoisomers were similar, except that (+)-neoisomenthol and (+)-neomenthol exhibited a slightly decreased affinity (Figure 1H). Only the *L* value of (+)-neomenthols was significantly reduced as compared to that of (−)-menthol (Figure 1I).

In contrast, at −80 mV, the maximum current activated by (+)-neoisomenthol and (+)-neomenthol was much reduced (Figure 1J), with their concentration–dependence curves largely shifted to higher concentrations (Figure 1K). As a result, the *K*_d_ values of stereoisomers were slightly but significantly increased, while their *L* values were much reduced (Figures 1L, M). The increase in *K*_d_ for (+)-menthol at −80 mV is more apparent as compared to the increase in other stereoisomers. We reason that such an increase in *K*_d_ for (+)-menthol is due to the relatively large *P*_o__max (therefore the smaller decrease in *L* value) induced by (+)-menthol at −80 mV (Figure 1K, lines in yellow and black, respectively). These observations clearly suggest that the less intense cooling sensation and higher cooling thresholds of the stereoisomers (Barel et al., 2009), such as (+)-neoisomenthol as compared to (−)-menthol, are due to both reduced binding affinity and ability in opening TRPM8 channel, especially at the more physiologically relevant −80 mV. Moreover, the further decreased gating capability (*L* values) of the stereoisomers at −80 mV as compared to those measured at +80 mV (Figures 1I, M, respectively) indicated that the ligand–protein interactions were modulated by transmembrane voltage.

### Interactions Between TRPM8 and the Stereoisomers Revealed by TMCA

To understand the origin of the differential activation of TRPM8 by the stereoisomers, we performed TMCA with patch-clamp recordings. Previously, we have employed this strategy to study the binding of (−)-menthol by replacing its hydroxyl group with an oxygen atom (the menthol analog menthone) or its isopropyl group with a methylethenyl group (the menthol analog isopulegol) (Xu et al., 2020). However, by using the menthol analogs, the chemical identity of a functional group in menthol was altered. To keep the chemical identity of functional groups and introduce perturbation of the chemical structure of menthol for TMCA, we employed the stereoisomers of menthol.

**FIGURE 2:**
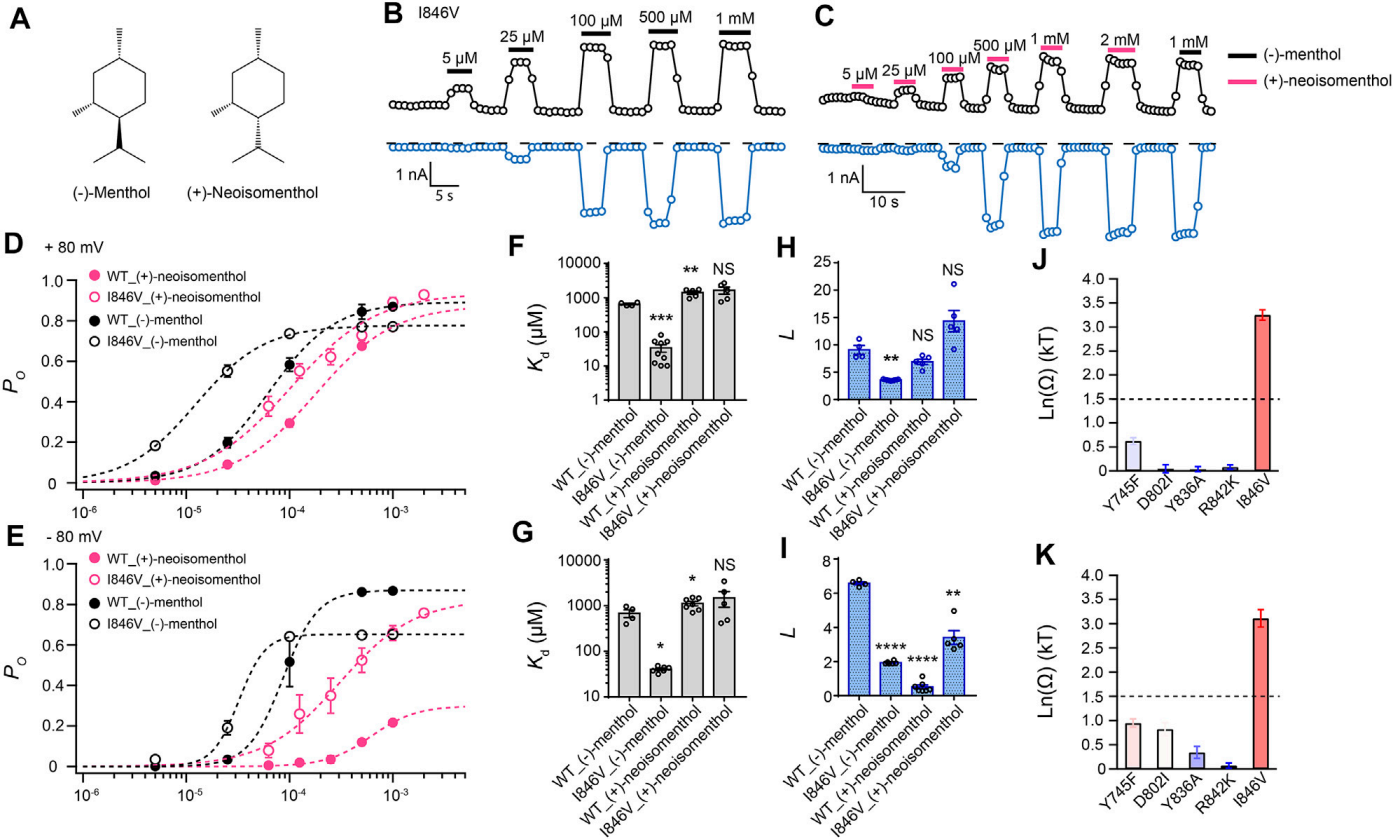
Differential interactions between mouse TRPM8 and (−)-menthol or (+)-neoisomenthol revealed by TMCA. **(A)** Comparison of chemical structures of (−)-menthol and (+)-neoisomenthol is shown. **(B)** Representative whole-cell recordings of the I846V mutant channel activated by (−)-menthol in a concentration-dependent manner. **(C)** Representative whole-cell recordings of the I846V mutant channel by (+)-neoisomenthol in a concentration-dependent manner. **(D)** Concentration–response curves of wild type and mutant such as I846V with either (-)-menthol or (+)-neoisomenthol activation were measured from whole-cell patch-clamp recordings at +80 mV (*n* = 5–9). **(F)** and **(H)** For wild type and I846V channel, *K*_d_ and *L* values at +80mV were calculated from the concentration–response curves in **(D)** (two-sided *t*-test, *p < 0.05; **p < 0.01; ***p < 0.001; ****p < 0.0001; NS, not statistically significant). **(J)** Summary of coupling energy measurements at +80 mV. Coupling energy was calculated from the *K*_d_ values. Mutants showing a coupling energy larger than 1.5 kT (dashed line) were colored in red. Those with less energy were colored in different shades of blue. At least four independent trials were performed for each chemical at each concentration. **(E)** Concentration–response curves of wild type and mutant such as I846V with either (−)-menthol or (+)-neoisomenthol activation were measured from whole-cell patch-clamp recordings at −80 mV (*n* = 5–9). **(G)** and **(I)** For wild type and I846V channel, *K*_d_ and *L* values at -80 mV were calculated from the concentration–response curves in **(E)** (two-sided *t*-test, *p < 0.05; **p < 0.01; ***p < 0.001; ****p < 0.0001; NS, not statistically significant). **(K)** Summary of coupling energy measurements at -80 mV. Coupling energy was calculated from the *K*_d_ values. Mutants showing a coupling energy larger than 1.5 kT (dashed line) were colored in red. Those with less energy were colored in different shades of blue. At least four independent trials were performed for each chemical at each concentration.

By comparing (−)-menthol and (+)-neoisomenthol (Figure 2A), we observed that they are only different in the orientation of the isopropyl group, while the special orientations of hydroxyl and methyl groups are identical. So these two stereoisomers are well-suited for probing the interaction between the isopropyl group and channel protein. By further measuring current activation by either (−)-menthol or (+)-neoisomenthol in WT TRPM8 and mutants like the I846V at ± 80 mV (Figures 2B,C), we first established the concentration dependence of channel open probability at either +80 mV or –80 mV (Figures 2D,E), and then calculated the corresponding *K*_d_ (Figures 2F,G) and *L* (Figures 2H,I) values of the stereoisomers based on their concentration–dependence curves. We further calculated the coupling energy of different mutants and observed that there is a large coupling (Figures 2J,K. 3.25 ± 0.11 kT and 3.10 ± 0.18 kT for +80 mV and – 80 mV, respectively) between the isopropyl group of menthol stereoisomers and residue I846 in the S4 of TRPM8. This is consistent with our previous findings that the isopropyl group of (-)-menthol interacts with L843 and I846 in TRPM8 (Xu et al., 2020). Moreover, the coupling energy values measured at either +80 mV or –80 mV were similarly large, indicating that though the S4 serves as, at least partially, a voltage sensor in TRPM8 channel (Voets et al., 2007), the interaction between the isopropyl group and TRPM8 is not affected by the transmembrane potential.

**FIGURE 3:**
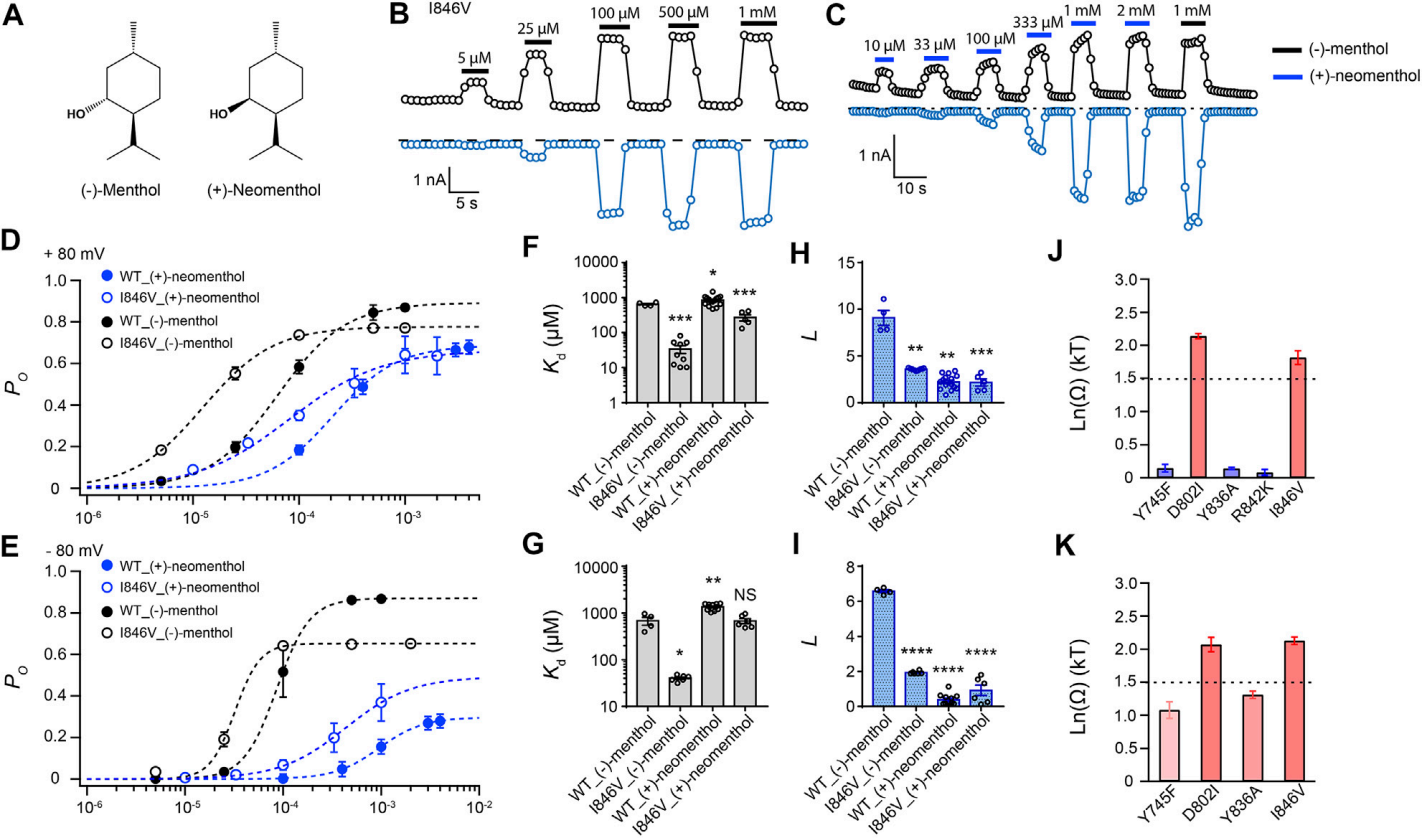
Differential interactions between mouse TRPM8 and (−)-menthol or (+)-neomenthol revealed by TMCA. **(A)** Comparison of chemical structures of (−)-menthol and (+)-neomenthol is shown. **(B)** Representative whole-cell recordings of the I846V mutant channel activated by (−)-menthol in a concentration-dependent manner. **(C)** Representative whole-cell recordings of the I846V mutant channel activated by (+)-neomenthol in a concentration-dependent manner. **(D)** Concentration–response curves of wild type and mutant such as I846V with either (−)-menthol or (+)-neomenthol activation were measured from whole-cell patch-clamp recordings at +80 mV (*n* = 5–9). **(F)** and **(H)** For wild type and I846V channel, *K*_d_ and *L* at +80 mV values were calculated from the concentration–response curves in **(D)** (two-sided *t*-test, *p < 0.05; **p < 0.01; ***p < 0.001; ****p < 0.0001; NS, not statistically significant). **(J)** Summary of coupling energy measurements at +80 mV. Coupling energy was calculated from the *K*_d_ values. Mutants showing a coupling energy larger than 1.5 kT (dashed line) were colored in red. Those with less energy were colored in different shades of blue. At least four independent trials were performed for each chemical at each concentration. **(E)** Concentration–response curves of wild type and mutant such as I846V with either (−)-menthol or (+)-neomenthol activation were measured from whole-cell patch-clamp recordings at −80 mV (*n* = 5–9). **(G)** and **(I)** For wild type and I846V channel, *K*_d_ and *L* values at -80 mV were calculated from the concentration–response curves in **(E)** (two-sided *t*-test, *p < 0.05; **p < 0.01; ***p < 0.001; ****p < 0.0001; NS, not statistically significant). **(K)** Summary of coupling energy measurements at −80 mV. Coupling energy was calculated from the *K*_d_ values. Mutants showing a coupling energy larger than 1.5 kT (dashed line) were colored in red. Those with less energy were colored in different shades of blue. At least four independent trials were performed for each chemical at each concentration.

To investigate the interaction between the hydroxyl group of the stereoisomers and TRPM8, we measured current activation by either (−)-menthol or (+)-neomenthol in WT TRPM8 and mutants like the I846V at ± 80 mV (Figures 3A–C), as only the hydroxyl group in these two stereoisomers differs in spatial orientation. We also determined the concentration dependence of channel open probability at either +80 mV or –80 mV (Figures 3D,E), and then calculated the corresponding *K*_d_ (Figures 3F,G) and *L* (Figures 3H,I) values of the stereoisomers based on their concentration–dependence curves. We further calculated the coupling energy of different mutants and observed that there is a large coupling (Figures 3J,K. 1.81 ± 0.10 kT and 2.12 ± 0.06 kT for +80 mV and –80 mV, respectively) between the hydroxyl group of menthol stereoisomers and residue D802 in the S3 of TRPM8. We also found that at -80 mV, (+)-neomenthol cannot activate the R842K mutant, preventing the determination of coupling energy at this residue. Our previous study detected large coupling energy between the hydroxyl group of (−)-menthol and D802 or R842 in TRPM8 by using the menthol analog menthone in TMCA (Xu et al., 2020), which is consistent with our observations.

Interestingly, we observed that the coupling energy at I846 was also larger than the 1.5 kT threshold, indicating that the orientation of the hydroxyl group may also affect how the isopropyl group interacts with the channel protein.

### Putative Binding Configurations of the Stereoisomers Suggested by Docking

**FIGURE 4:**
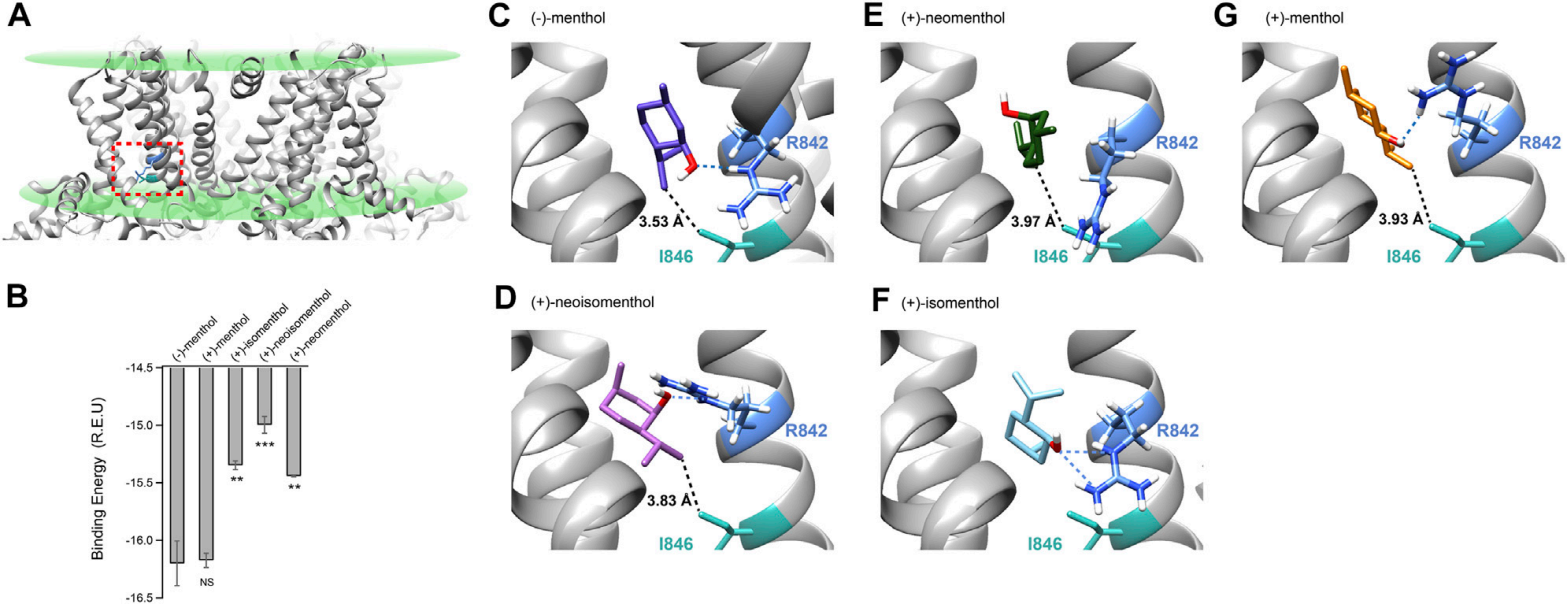
Molecular docking suggested that menthol stereoisomers activated mouse TRPM8 in different binding configurations. **(A)** Putative menthol-binding pocket located within the transmembrane domains of the TRPM8 channel as revealed by cryo-EM in apo state (PDB ID: 6BPQ). R842 and I846 are highlighted in blue and green, respectively. **(B)** Menthol stereoisomers were docked into the binding pocket. Averaged binding energy of the top ten binding models of each stereoisomer was measured. All statistical data are given as mean ± s.e.m. (*n* = 10, two-sided *t*-test, *p < 0.05; **p < 0.01; ***p < 0.001; ****p < 0.0001; NS, not statistically significant). **(C–G)** Docking of menthol stereoisomers into the binding pocket. The hydroxyl group of stereoisomers was predicted to form a hydrogen bond with sidechain R842 (dashed line in blue), and the distance of the isopropyl group with sidechain I846 is shown with dashed lines in black.

As the menthol-bound state of TRPM8 has not been directly revealed by cryo-EM despite the fact that several TRPM8 structures have been reported (Yin et al., 2018; Diver et al., 2019; Yin et al., 2019), we employed molecular docking in the Rosetta suite (Leaver-Fay et al., 2011) to investigate the possible binding configurations of menthol stereoisomer. We computationally docked (−)-menthol, (+)-neoisomenthol, (+)-neomenthol, or (+)-isomenthol into the pocket formed by the S1 to S4 transmembrane helices in the apo state of TRPM8 (Figures 4A,B, dashed box in red, PDB ID: 6BPQ) because this site has been validated as the menthol binding pocket in previous studies (Bandell et al., 2006; Xu et al., 2020). We then performed a statistical analysis of the docking models by plotting their binding energy against the rmsd of the best scoring models (Supplementary Figures S1C–G). The docking models exhibited a funnel-shaped distribution of binding energy, supporting the validity of the docking results. Moreover, we performed cluster analysis (Supplementary Figures S1H–L). We observed that the docking models we originally showed in Figure 4 are indeed clustered with a converged ligand binding configuration among the top 10 binding energy models. Specifically, the cluster of the representative (-)-menthol model shown in Figure 4 contained 9 out of the top 10 models (Supplementary Figure S1H). The cluster of representative (+)-menthol, (+)-isomenthol, (+)-neoisomenthol, and (+)-neomenthol contained 3, 6, 4, and 6 models among the top 10 models, respectively. Therefore, the representative docking models shown in Figure 4 are reliable.

To validate our docking results, we performed a molecular dynamics simulation as suggested by the reviewer (see Methods for details). Starting from our (−)-menthol docking model, the S1–S4 domains remained stable during the 378 ns simulation with RMSD being around 3 Å (Supplementary Figure S2A). The menthol molecule is bound stably within its binding pocket inside the S1–S4 domains, as illustrated in the ensemble plot of the menthol molecule with snapshots of the simulation from the beginning to the end (Supplementary Figure S2B, red and blue, respectively; Supplementary Movie S1). The distance between the oxygen atom in menthol and the OD atom of D802, as well as the distance between the C9 atom in menthol and the CD atom of I846, remained stable during the 378 ns simulation, indicating the docking configuration of menthol is stable.

Our previous study has shown that for (−)-menthol, its hydroxyl group forms a hydrogen bond with the sidechain of R842, while the isopropyl group is within 4 Å of the sidechain of I846 (Figure 4C, dashed lines in blue or black, respectively) (Xu et al., 2020). For (+)-neoisomenthol, which differs from (-)-menthol in the orientation of the isopropyl group (Figures 1A,D), its hydroxyl group also formed a hydrogen bond with the sidechain of R842 (Figure 4D, dashed line in blue). Though the isopropyl group of (+)-neoisomenthol points in the opposite direction as compared to that in (−)-menthol, it was still in contact with the sidechain of I846 (3.83 Å, Figure 4D, dashed line in black). These observations suggested that the “grab and stand” binding mechanism we established in (−)-menthol (Xu et al., 2020) is also applicable to (+)-neoisomenthol, validating the use of TMCA as these two stereoisomers shared similar binding configurations. Moreover, the binding energy of the top 10 (+)-neoisomenthol docking models (−14.99 ± 0.07 Rosetta Energy Unit (R.E.U.)) was predicted to be smaller than that of (−)-menthol (−16.20 ± 0.19 R.E.U.) (Figure 4B), which was consistent with the increased *K*_d_ values measured from patch-clamp recordings (Figures 1H, L). The upward-pointing sidechain of R842 upon (+)-neoisomenthol binding as compared to the downward-point conformation in (−)-menthol bound model may offer clues to understand the reduced channel opening capability (reduced *L* values measured from patch-clamp recordings) of (+)-neoisomenthol (Figure 1M).

However, when (+)-neomenthol, which differs from (−)-menthol in the orientation of hydroxyl group (Figures 1A,E), was docked into TRPM8, the models with the top 10 largest binding energy were converged into a different configuration (Figure 4E). The orientation of hydroxyl group in (+)-neomenthol pointed away from the sidechain of R842, so that the hydrogen bond between the hydroxyl group of (-)-menthol and R842 was disrupted. Indeed, the binding energy of (+)-neomenthol was significantly smaller than that of (-)-menthol (Figure 4B), which is consistent with the increased *K*_d_ values measured from patch-clamp recordings (Figures 1H, L). As R842 was no longer “grabbed” by the hydroxyl group, the channel opening capability (reflected in *L* values) of (+)-neomenthol was likely reduced (Figures 1I, M). Furthermore, as the isopropyl group of (+)-neomenthol pointed away from I846 with an increased distance of 3.97 Å (Figure 4E, dashed line in black), the interaction between the isopropyl group and I846 was most likely perturbed, so that it is not surprising that we observed large coupling energy values between (+)-neomenthol and I846 (Figures 3J,K).

Furthermore, we also docked (+)-isomenthol or (+)-menthol into TRPM8. As these two stereoisomers contain either two or three chemical groups with distinct spatial orientations compared to (-)-menthol, it is not feasible to perform TMCA by comparing (-)-menthol and these ligands to directly probe molecular interactions with TRPM8. Nevertheless, from docking, we gained insights into their binding and activation of the channel. For (+)-isomenthol, the top 10 docking models with the largest binding energy well converged into a configuration where the isopropyl group pointed upward away from I846, while the hydroxyl group still formed a hydrogen bond with the sidechain of R842 (Figure 4F, dashed lines in blue). The reduced binding energy of (+)-isomenthol (Figure 4B) was consistent with the increased *K*_d_ measured at -80 mV (Figure 1L).

For (+)-menthol, though its chemical groups differ from (−)-menthol in all three chiral centers, the docking figuration was similar to that of (−)-menthol. Its hydroxyl group formed a hydrogen bond between the sidechain of R842 and its isopropyl group pointed toward I846 (Figure 4G, dashed line in blue and black, respectively). Such a similar binding configuration of (+)-menthol was in line with its unchanged binding energy predicted from docking (Figure 4B) and *K*_d_ measured at +80 mV (Figure 1H), though *K*_d_ and *L* measured at −80 mV were still significantly changed as compared to (−)-menthol (Figures 1L, M).

## Discussion

In this study, we systematically investigated how the five commercially available stereoisomers of menthol bind and activate the TRPM8 channel with patch-clamp recordings and molecular docking. We observed that (−)-menthol, which is the most abundant menthol stereoisomer in mints, best activated the TRPM8 channel with the largest *P*_o__max (therefore the largest *L* value) and the lowest *K*_d_, while (+)-menthol exhibited slightly altered current activation properties (Figure 1). Such a similarity in TRPM8 activation by (−)-menthol and (+)-menthol could be explained by their similar putative binding configuration, where the hydroxyl group “grabs” the D802/R842 with a hydrogen bond and the isopropyl group “stands on” I846 (Figure 4). In contrast, (+)-isomenthol, (+)-neoisomenthol, and (+)-neomenthol showed significantly reduced *P*_o__max (therefore smaller *L* values) and increased *K*_d_, especially at −80 mV (Figure 1), which is likely due to altered binding configurations where the “grab and stand” mechanism is disrupted (Figure 4). Therefore, our observations lead to mechanistical insights regarding the differential activation of TRPM8 by the menthol stereoisomers.

TMCA by patch-clamp recording has been widely used to probe ligand-protein interactions. For instance, this approach was employed to study how peptide toxin bind to the outer pore of voltage-gated potassium channel (Ranganathan et al., 1996) and TRPV1 channel (Yang et al., 2017). TMCA is applicable to investigate how small molecules such as capsaicin (Yang et al., 2015) or (−)-menthol (Xu et al., 2020) interact with TRP channels. However, we observed that when the ligand is small in a chemical structure like menthol, altering one chemical group of the ligand may affect how another group interacts with the protein. When the orientation of the hydroxyl group in (−)-menthol is changed as in (+)-neomenthol, we observed that besides the hydrogen-bounding D802 residue, I846 residue also showed large coupling energy (Figures 3J,K). We reason that most likely in (+)-neoisomenthol, its overall binding configuration was slightly changed so that how its isopropyl group interacted with I846 was accordingly altered. Indeed, our docking results suggested that the distance between the isopropyl group of (+)-neoisomenthol and the sidechain of I846 was increased as compared to that of (−)-menthol (Figures 4C,D). Therefore, caution must be taken regarding the interpretation of coupling energy values measured from TMCA for small molecules.

TRP channels like TRPM8, are polymodal receptors modulated by a plethora of physical and chemical stimuli (Julius, 2013; Zheng, 2013), including the transmembrane voltage. For TRPM8 activation by the stereoisomers of menthol, we clearly found that the *P*_o_ _max of TRPM8 (therefore the *L* values of the ligands) was reduced at a hyperpolarized voltage (−80 mV) as compared to that at depolarization (+80 mV) (Figures 1G,K). For (+)-neoisomenthol and (+)-neomenthol, such a reduction in *P*_o_ _max was even larger, which may explain the less intense cooling sensation elicited by (+)-neoisomenthol because the physiologically relevant transmembrane voltage resides within the hyperpolarized range (Hopp, 1993; Hille, 2001; Barel et al., 2009). Mechanistically, given menthol interacts with the voltage-sensing residue R842 (Voets et al., 2007; Xu et al., 2020) in S4 or D802 in S3 (Figures 3J,K), it is not surprising that the menthol activation is drastically affected by voltage. However, because the cryo-EM structures of TRPM8 channel were determined at zero transmembrane voltage, they do not represent the deactivated structural state of the voltage sensing S1 to S4 domains. Therefore, it will require more work in the future to deduce the structural basis for the voltage modulation of the menthol stereoisomer binding and activation of TRPM8.

## Data Availability

The original contributions presented in the study are included in the article/Supplementary Material; further inquiries can be directed to the corresponding author.

## Supplementary Material

Supplementary Figure S1 Binding energy contribution from menthol stereoisomers docking results. **(A)** Alignment of the amino acid sequence of S1–S4 in murine TRPM8 (mTRPM8) and human TRPM8 (hTRPM8). The S1 to S4 regions (wave line in blue) are indicated. The protein sequence between mTRPM8 and hTRPM8 is highly conserved with the identity of 96.61%. **(B)** Representative whole-cell recordings showed that (−)-menthol activated mTRPM8 at ± 80 mV. **(C–G)** Menthol stereoisomers docking models exhibited a funnel-shaped distribution of binding energy versus the ligand RMSD as calculated by Rosetta (R.E.U., Rosetta energy unit). The top one models with the lowest binding energy, which was the docking model presented in Figure 4, were highlighted in red. **(H–L)** Docking models with the best binding scores are clustered in a converged configuration. The cluster of representative (−)-menthol, (+)-menthol, (+)-isomenthol, (+)-neoisomenthol, and (+)-neomenthol contained 9, 3, 6, 4, and 6 models among the top ten models, respectively.

Supplementary Figure S2 Molecular dynamic analysis of TRPM8 and menthol ligand. **(A)** RMSD of S1–S4 domains calculated by molecular dynamic stimulation during 378 ns is around 3 Å. **(B)** Ensemble plot of (**–**)-menthol molecule during stimulation. The configuration from the beginning is shown in red, and the configuration at the end is shown in blue. **(C)** Distance between specific atoms of (**–**)-menthol and residues is displayed. The distance between the oxygen atom in menthol and the OD atom of D802 is shown in blue line, and the distance between the C9 atom in menthol and CD atom of I846 is shown in gray line.
