# Supplementary material for: Differential Activation of TRPM8 by the Stereoisomers of Menthol
Source: Front Pharmacol. 2022 Jun 21;13:898670. doi: 10.3389/fphar.2022.898670 (PMC9253294; doi:10.3389/fphar.2022.898670)
Supplement: Supplementary file 1 [file Table1.docx]

**Supplementary Table 1. Primers used in this study to generate point mutations.**

| Name | Primer-Forward | Primer-Reverse |
| --- | --- | --- |
| Y745F | CGTGGTCTTCTTCATCGCCTTCCTCCTGCTGTTTGCCT | GGAAGGCGATGAAGAAGACCACGTTCCAGGAGAAGACC |
| D802I | GAACGTTATGATCACCCTGGGACTCTTCTACTTCATAG | GTCCCAGGGTGATCATAACGTTCCATAGGTCGGTGAAA |
| Y836A | CTGTCTGGATGCCATTATATTCACGCTAAGGCTCATCCAC | GAATATAATGGCATCCAGACAGAAAATGACGCGC |
| R842K | ATTCACGCTAAAGCTCATCCACATTTTCACCGTCAGCA | TGTGGATGAGCTTTAGCGTGAATATAATGTAATCCAGA |
| I846V | GCTCATCCACGTTTTCACCGTCAGCAGGAACTTGGGAC | TGACGGTGAAAACGTGGATGAGCCTTAGCGTGAATATA |
