# Supplementary figures and images for: Differential Activation of TRPM8 by the Stereoisomers of Menthol
Source: Front Pharmacol. 2022 Jun 21;13:898670. doi: 10.3389/fphar.2022.898670 (PMC9253294; doi:10.3389/fphar.2022.898670)

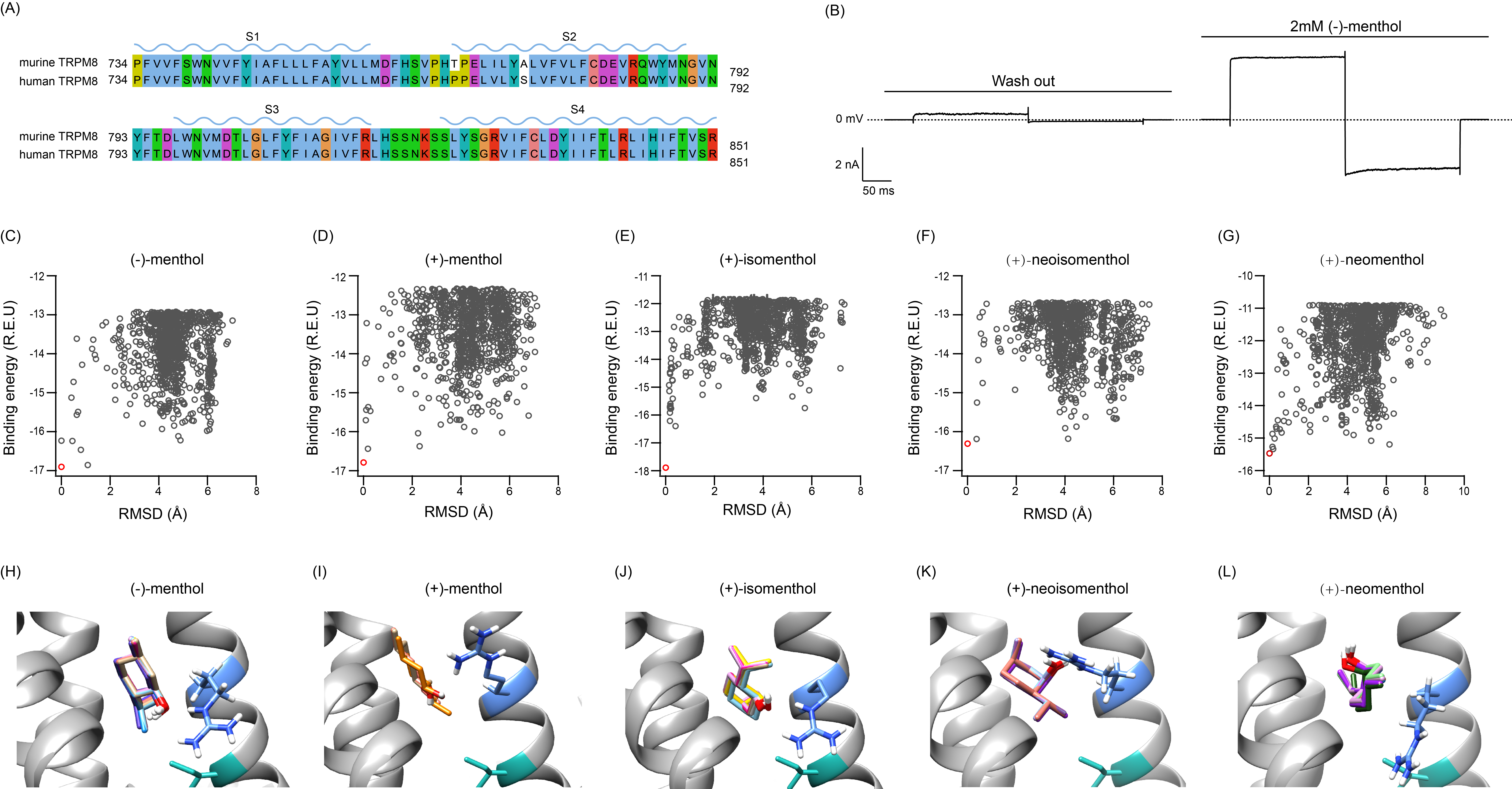

Supplement: Supplementary file 2 [file Image1.JPEG]

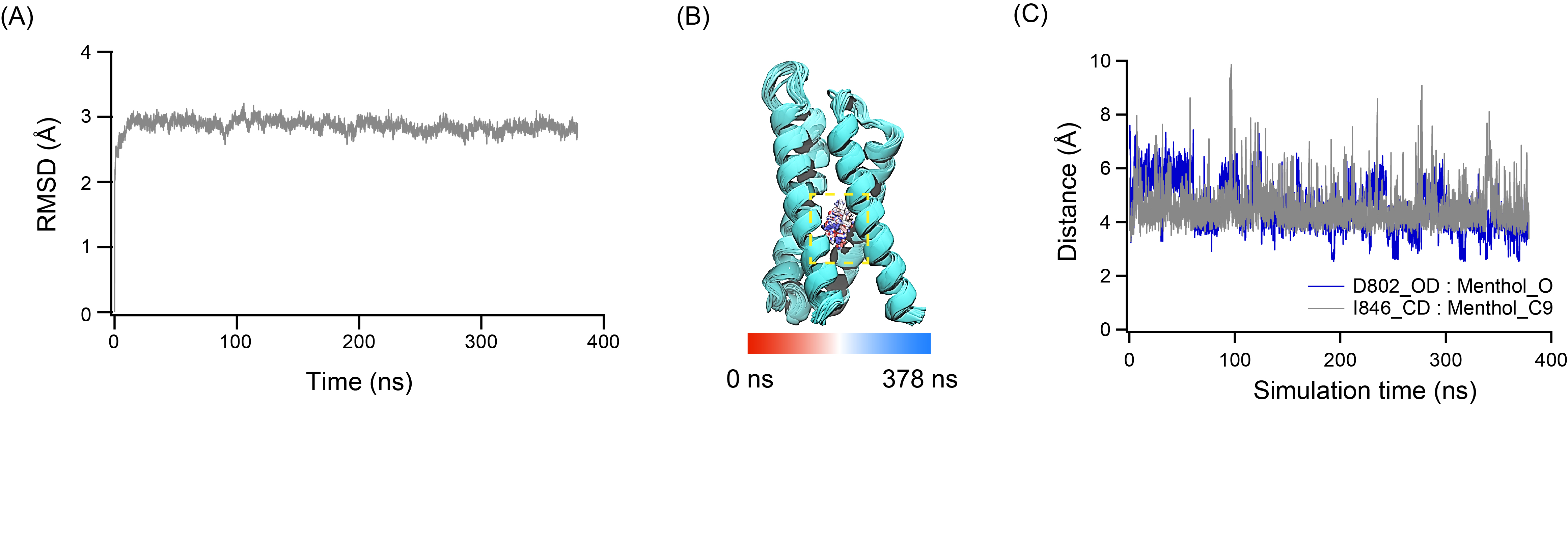

Supplement: Supplementary file 3 [file Image2.JPEG]
